# Cognitive reappraisal as a component of process-based misophonia treatment: a mixed-methods pilot study of feasibility, acceptability and initial outcomes

**DOI:** 10.3389/fpsyt.2026.1744882

**Published:** 2026-06-24

**Authors:** Marta Siepsiak, Andrzej Śliwerski, Anna Turek-Wojnarowicz, Weronika Araszkiewicz, Małgorzata Gambin, Mark Zachary Rosenthal

**Affiliations:** 1 Department of Psychology, SWPS University in Warsaw, Warsaw, Poland; 2 Institute of Psychology, University of Łódź, Łódź, Poland; 3 Faculty of Psychology, University of Warsaw, Warsaw, Poland; 4 Department of Psychiatry & Behavioral Sciences, Duke University, Durham, NC, United States; 5 Department of Psychology & Neuroscience, Duke University, Durham, NC, United States

**Keywords:** cognitive reappraisal, decreased sound tolerance, feasibility & acceptability, misophonia, pilot study, process-based treatment, psychological intervention, psychotherapy

## Abstract

**Objective:**

Misophonia is a subtype of decreased sound tolerance associated with significant distress and reduced quality of life. Although no evidence-based treatments exist specifically for misophonia, cognitive behavioral therapies (CBTs) show promise. Cognitive reappraisal (CR), a core transdiagnostic CBT intervention, is frequently used in experimental studies, however, its effects on misophonia in ecologically valid conditions remain untested. Similarly, the needs and perspectives of individuals with misophonia regarding treatment are underexplored. This study developed and evaluated a CR protocol tailored for misophonia, assessing feasibility, acceptability and satisfaction, as well as preliminary assessment of the intervention outcomes through an uncontrolled quantitative as well as qualitative design.

**Methods:**

The intervention included a 90-minute group session and three 30-minute individual online sessions over four weeks. Adults (*N* = 23) were recruited using structured interviews. Misophonia symptoms were assessed at two pre-treatment time points and post-treatment. Semi-structured group interviews explored participants’ experiences.

**Results:**

No significant changes were observed between the two pre-treatment assessments in three of the five symptom subscales, partially confirming baseline symptom stability. In contrast, a significant post-treatment reduction in externalizing responses to misophonia triggers was observed (*Estimate* = -8.47, SE = 2.32, *t*(42.45) = -3.65, *p* < 0.001), with similar improvements across other S-Five subscales. Qualitative data highlighted feelings of isolation, appreciation for the group format, and a need for more individualized support.

**Conclusion:**

CR shows promise as a component of misophonia treatment that warrants further controlled evaluation. The participant’s feedback highlights the importance of tailoring interventions to individual needs and patient perspectives.

## 1 Introduction

Misophonia is a relatively newly named disorder defined by longstanding functional impairment and/or psychological distress in response to common repetitive sounds, usually featuring oral/facial noises made by others (e.g., chewing, throat-clearing, heavy breathing, tapping; 1)⁠. Studies suggest that it appears to typically develop in childhood or adolescence (2, 3)⁠ and may persist well into adulthood. The estimated prevalence of clinically elevated levels of misophonia in large representative samples ranges between 2–4% in Germany (4, 5) to 4.6% in the United States (6). Although misophonia is not yet included in the International Classification of Diseases (ICD; though a proposal is currently under review by ICD-11), numerous studies and clinical data indicate clear and distinguishable symptoms of the condition (1, 7–11)⁠.

Specific triggers and symptoms of misophonia can vary significantly among individuals. For example, differences may exist in the intensity of autonomic nervous system hyperarousal (e.g., heart racing, sweating, muscle tension), attentional dysfunction (e.g., distractibility, hypervigilance), externalizing (e.g., They are rude, they should not behave like that) versus internalizing attributions (e.g., I am a bad person, I should not feel such emotions in response to sounds) related to triggers (12, 13)⁠, the extent to which individuals avoid triggers, use of varied problematic coping responses to triggers (e.g., 14–16). As such, a single one-size-fits-all approach is unlikely to be successful.

While there are no controlled clinical studies supporting a purely sound-based audiological treatment for misophonia, interventions within the family of CBTs, including third-wave approaches (17)⁠, have emerged as the most researched and promising interventions in the field (12, 18, 19) Systematic review of psychological interventions can be found in Mattson et al. (20) and 21.

Cognitive reappraisal (CR) is one of the most widely used evidence-based psychotherapeutic interventions (22–26)⁠. It is designed to improve functioning and reduce emotional distress by identifying and changing unhelpful patterns of thinking in response to certain classes of stimuli. Decades of laboratory and clinical research support the efficacy of CR for children and adults across a wide range of mental and physical health problems (for comprehensive reviews, see 27–29).

Preliminary research indicates that CR may be effective in addressing misophonia symptoms. Siepsiak et al. (30)⁠ experimentally demonstrated that sounds typically perceived as triggering can evoke less negative reactions when presented as something else (e.g., eating sounds visually depicted as hands playing in mud). Similar findings were reported by Heller & Smith (31)⁠, who showed that misidentification of a sound influences its pleasantness rating, making potential trigger sounds less aversive. Savard et al. (32) also obtained comparable results and further demonstrated that the effect is stronger in individuals with higher misophonia symptoms, who rated sounds - especially trigger sounds - as significantly more aversive once they became identifiable. Moreover, Samermit et al. (33) found that certain sounds, when paired with incongruent but non-aversive visual stimuli (e.g., chewing sounds paired with a video of someone stepping on snow), were perceived as more pleasant. While these findings lay the groundwork for developing reappraisal-based treatments for misophonia sufferers, they did not specifically investigate intentional use of reappraisal as an emotional regulation strategy.

Efforts to alter the response to misophonia triggers by changing the context in which they are experienced have been explored in therapeutic interventions (e.g., 12, 19, 34–36)⁠⁠. However, CR was either not singled out as a specific strategy or was one intervention component integrated with numerous other therapeutic techniques, complicating the assessment of its individual impact and efficacy. More recently, Neacsiu et al. (37)⁠ published a study in which cognitive restructuring was used in both the misophonia group and the group of individuals with general emotional dysregulation. Although the study yielded promising results, it was experimental and conducted in a highly controlled laboratory setting, which limits generalizability to the use of CR in psychotherapy and underscores how early we still are in parsing specific mechanisms. Therefore, in this study, we adapted the core elements of Neacsiu et al.’s (37) protocol, making modifications to certain sections and adding new components to better suit our study’s goals. We also modified the study protocol to allow for greater flexibility and individualization, enhancing its ecological validity, for example, by adding three individual sessions to the group-session protocol. The full set of materials—including the protocols revised after the pilot study and the CR Guide developed within this project (also provided to participants)—will be published separately and are available from the authors upon reasonable request. The cheat sheet, adopted without modification from Neacsiu et al. (37), is not included here, and the CR Quiz used in that study was not implemented in our protocol.

While comprehensive, multi-component interventions like traditional CBT (19) and the Unified Protocol (35) have demonstrated broad clinical utility, isolating specific mechanisms remains important. This pilot study adds to the existing literature by providing a preliminary, narrow focus on CR to better understand its specific role and feasibility as just one potential active ingredient within these broader process-based treatments for misophonia sufferers.

Despite the growing body of knowledge on CBTs for the treatment of misophonia, the experiences of individuals with this disorder in the context of therapeutic interventions, including CR, remain largely unexplored. This study aims to address these gaps by conducting a preliminary evaluation of participants’ lived experiences with treatment. To better understand and learn from participants’ experiences and to use this feedback to inform protocol modifications for future studies and clinical applications, we used Reflexive Thematic Analysis (38–40)⁠. A similar approach has been used in previous studies to explore experiences with physical activity counseling in people with depression (41)⁠, rehabilitation in individuals suffering from chronic pain (42)⁠, and CBT-based interventions for parents of children with chronic pain (43)⁠.

In sum, to our knowledge, this is among the first studies to carefully investigate CR as a standalone intervention component for misophonia in a real-world, non-laboratory setting. Designed as a formative evaluation (44, 45) in preparation for a fully powered Randomized Controlled Trial (RCT), this mixed-methods pilot study primarily aimed to assess the feasibility and acceptability. Specifically, we sought to verify treatment satisfaction, estimate retention and dropout rates, and gather qualitative feedback from participants to identify necessary modifications to the protocol. Moreover, we aimed to conduct an exploratory assessment of preliminary clinical outcomes to observe initial trends in symptom reduction.

## 2 Methods

The study was approved by the Research Ethics Committee (approval no. 14/11/2023/26). Electronic informed consent was obtained from all participants. This mixed-methods pilot study was conducted to evaluate the feasibility and acceptability of procedures for an ongoing parent randomized controlled trial (RCT; ClinicalTrials.gov: NCT06372405) (46). The general structure of the study was based on the parent RCT protocol, with several key differences tailored to our pilot objectives (e.g., the absence of a control group and the inclusion of qualitative analyses not conducted in the RCT).

### 2.1 Participants

Twenty-six participants initially enrolled in the study, of whom 23 completed the intervention and assessments, forming the final sample (*M*age = 32, *SD* = 8, range = 19–53). Misophonia symptom severity, as measured by the MisoQuest (see Assessment section), was *M* = 65 (*SD* = 4, range = 55–70). Four participants scored lower than the 61-point cutoff suggested by Siepsiak et al. (47), with scores of 60, 60, 56, and 55. All participants met the criteria for misophonia as assessed by the Duke Misophonia Interview (see Assessment section). A total of eight participants had a history of psychiatric disorders (as assessed by M.I.N.I, see Methods), three of whom met diagnostic criteria for at least one current disorder at the time of the study [current diagnoses included major depressive disorder (*n* = 2), panic disorder (*n* = 1), binge eating disorder (*n* = 1), and generalized anxiety disorder (*n* = 1)]. Past disorders or conditions included major depressive disorder (*n* = 6) and suicidal ideation (*n* = 2). Seven participants reported migraines. None of the participants met the criteria for any personality disorder as assessed by SCID 5-PD (six participants were assessed only with the OCPD module; see Assessment). A dimensional description of personality disorder traits is presented in **Table 1**, with obsessive-compulsive personality disorder (OCPD) traits showing the highest mean level (*M* = 2.43, *SD* = 2.81). Twelve participants reported onset of misophonia symptoms before age 9, eight participants between ages 10 and 14, and three participants between ages 15 and 18. In nine participants (39.1%), misophonia was reportedly also present in one of their family members.

**Table 1:** The severity of personality disorder traits, as measured by the SCID-5-PD.

Personality disorder	*N*	Min	Max	*M*	*SD*
Obsessive–Compulsive (OCPD)	23*	0	9	2.43	2.81
Schizoid	17	0	8	1.18	2.19
Avoidant	17	0	5	0.94	1.52
Dependent	17	0	5	0.47	1.37
Paranoid	17	0	4	0.29	0.99
Borderline	17	0	4	0.29	0.99
Antisocial	17	0	2	0.29	0.69
Schizotypal	17	0	2	0.24	0.56
Histrionic	17	0	0	0.00	0.00
Narcissistic	17	0	0	0.00	0.00

*A total of six participants did not participate in the on-site assessment and therefore could not complete the full SCID-5-PD interview. As we expected OCPD traits to be heightened in the misophonia group, and as this measure was required for another analysis beyond the scope of this study, these six participants were assessed using only the OCPD module. This resulted in a difference in the number of observations between OCPD and the other personality disorders.

Despite efforts to recruit without gender bias, only one male participated, and none of the study participants identified as non-binary. All participants self-identified as White, Polish-speaking, and of Polish nationality. The majority of participants had completed master degree (*n* = 13, 56.5%); four participants had a bachelor’s degree (17.4%), five had post-secondary education (21.7%), and one had secondary education. Most participants resided in large cities with populations over 500,000 (39.1%), in cities with populations over 100,000 (21.7%), and in smaller towns (17.4%).

### 2.2 Study procedure

The study was advertised via social media (Facebook), radio and newspapers. In the main advertisement post, we announced that we were searching for people aged 18 to 55 who could not tolerate eating or other oral sounds and were fluent in Polish. Exclusion criteria included current anorexia, manic and/or psychotic disorders, self-reported hearing impairment, heart disease or the presence of pacemaker, any mental or physical health issues that could endanger health or life within the next three months, and participation in psychotherapy within the last three months, as its techniques and outcomes could overlap with the study procedures. The exclusion criteria were highly specific (e.g., heart disease) because, in the randomized controlled trial (RCT), we additionally used psychophysiological measures to assess the impact of CR, and these criteria were kept identical in both the pilot and the RCT, as registered on ClinicalTrials.gov. Finally, requiring eating sounds as the primary trigger was necessary due to specific experimental stimuli that needed to be relatively consistent across participants (although the experimental component is beyond the scope of the current study).

**Figure 1:**
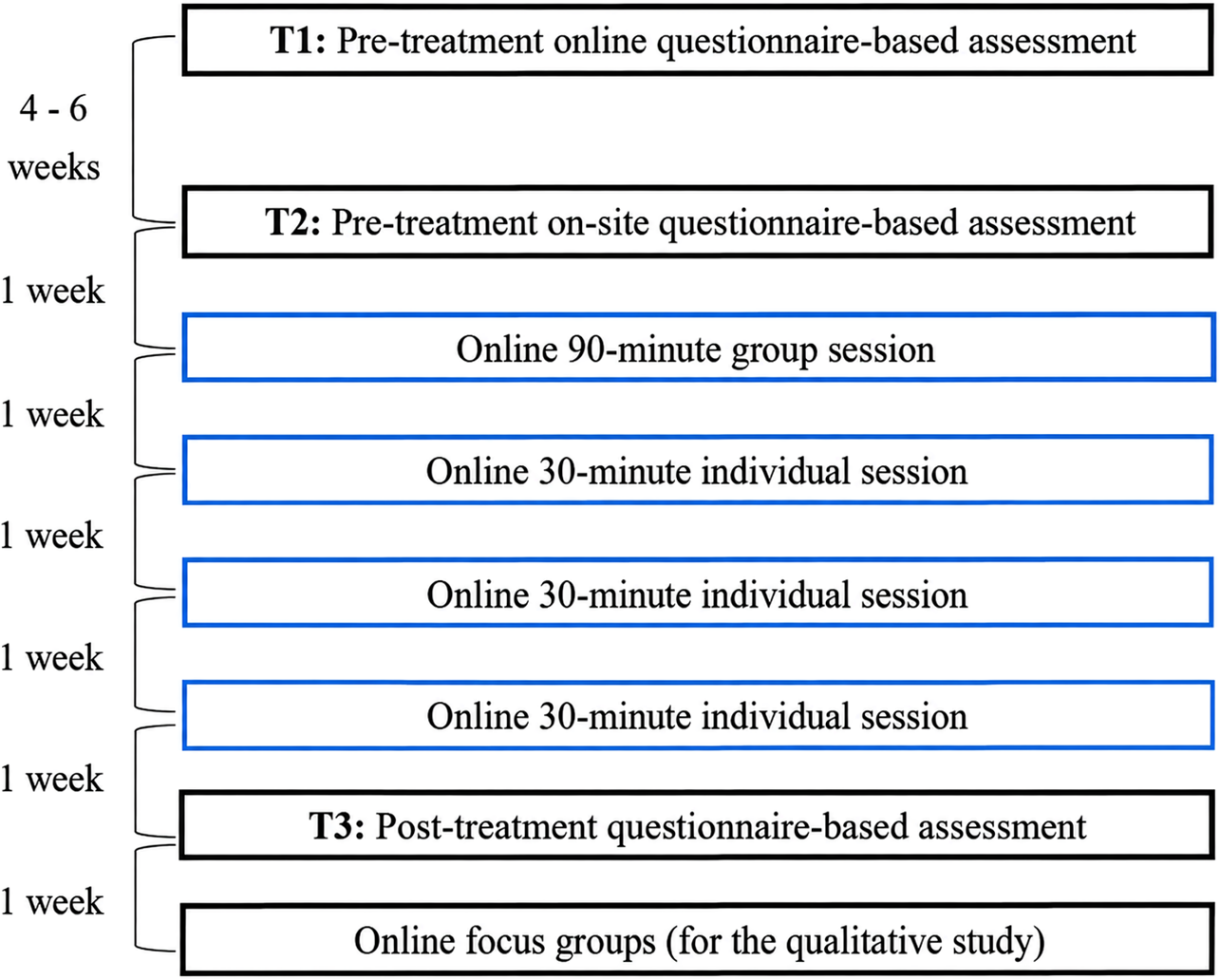
The structure of the study.

There were two stages in the recruitment process: online questionnaires and demographic data (described in the Assessment section under 1 and 2) and online, face-to-face semi-structured interviews (described in the Assessment section under 3, 4, and 5). Participants who met the eligibility criteria (see the Assessment) were invited to participate in the study. During the on-site visit they underwent a more comprehensive interview to evaluate psychiatric and personality disorders (described in the Assessment section under 4 and 6).The interval between the first (T1) and second (T2) pre-treatment assessments (7) was 4–6 weeks, followed by four weekly therapeutic sessions over a period of four weeks. Post-treatment assessment (T3) was conducted one week after the last session, which also included a treatment satisfaction survey (8). After completing the study, participants received an email with an invitation to take part in an audio-recorded meeting, where they could discuss their experiences with the project (9). Two focus groups took place one week after the last session. Participants were informed that this was an optional additional component, and that the interviews would be transcribed, analyzed, and the results published. The structure of the main study is presented in **Figure 1**.

### 2.3 Assessment

Although there is a consensus definition of misophonia (1), it is not yet recognized as a formal diagnosis with specific criteria in major classification systems. However, the consensus definition and virtually all studies suggest that oral sounds are among the most common triggers. As such, and to align with the experimental design of our parent study, which utilized eating sounds as stimuli, we included only participants whose main triggers were related to such sounds. Crucially, by combining this specific trigger criterion with validated assessment tools (the MisoQuest and the Duke Misophonia Interview), we ensured that our sample strictly aligns with misophonia as understood by the Swedo et al. (1) consensus, differentiating it from more general decreased sound tolerance or hyperacusis.

1. The MisoQuest (47)⁠ was used to screen for the severity of misophonia symptoms in order to identify individuals for the face-to-face interviews for final inclusion. The questionnaire was developed and validated in a sample of Polish-speaking participants. Each of the 14 items is rated on a 1–5 scale. Higher scores reflect greater severity of misophonia symptoms. Cronbach’s alpha (α) in this study was 0.83. Previous research indicates that the MisoQuest is highly specific but less sensitive, often yielding false negatives when a conservative cutoff is applied (48). Therefore, we used a lowered cutoff score of >48 points, instead of the initially suggested 61 points (indicating clinically significant misophonia symptoms). This decision aimed to minimize false negatives and maximize the pool of potential candidates, given the demanding nature of the study, which required participants to travel to Warsaw twice. Importantly, no participants were excluded based on the subsequent clinical interview and the lowest MisoQuest score in our final sample was 55.
2. The Misophonia Questionnaire (49) was used in the first stage of recruitment to identify the most disturbing sounds. Because eating sounds were required as the primary trigger, only the first part of the lists of triggers was used in this study.
3. The Duke Misophonia Interview (DMI; 14; Polish version: Siepsiak et al., manuscript in preparation)⁠ was used to confirm clinically significant misophonia symptoms, defined as symptoms that significantly impact the individual’s daily functioning.
4. The Mini-International Neuropsychiatric Interview, Standard Version 7.0.2 for DSM-5 and ICD-10 (M.I.N.I; 50; Polish version by Mapi Research Trust) was used to screen for current (hypo)manic episodes, anorexia, psychotic disorders, and current risk of suicidality. It was also used to assess coexisting disorders.
5. Additional questions were used to verify the inclusion and exclusion criteria. These included inquiries about heart disease, medication use, the presence of a pacemaker, participation in psychotherapy within the last three months, and hearing issues (such as notable hearing loss).
6. When necessary, further questions were used to assess the participant’s current mental state and evaluate the risk of crises or potential health deterioration during the study.
7. The Structured Clinical Interview for DSM-5 Personality Disorders (SCID-5-PD; 51) was used to assess personality disorder symptoms both categorically and dimensionally. First, participants completed a self-report screening questionnaire. Subsequently, based on their responses, they were interviewed face-to-face on-site. When appropriate, additional sections were administered based on the psychologists’ evaluation. Because six participants took part in the study exclusively online, without an on-site laboratory assessment, they were unable to complete the questionnaire and undergo the full SCID procedure. However, since OCPD had previously been suspected to be related to misophonia, we administered the OCPD module online to these participants (as the full SCID would have been too lengthy).
8. The Selective Sound Sensitivity Syndrome Scale (S-Five; 52; Polish validation: 53) was used to evaluate misophonic experience, identifying five aspects: Externalizing appraisals, Internalizing appraisals, Impact, Threat, and Outburst. Externalizing appraisals capture negative appraisals of others when they produce sounds, such as perceiving them as rude, disrespectful, or inconsiderate, and sometimes attributing intentionality. Internalizing appraisals reflect negative self-directed thoughts (e.g., feeling like a bad or angry person because of sound reactions, or feeling guilty for such reactions). Impact measures the perceived limitations caused by misophonia, both current and anticipated (e.g., avoiding places, missing social or work opportunities). Threat reflects feelings of distress, helplessness, and fear of being trapped when unable to escape sounds. Outburst assesses aggressive impulses or fear of losing control in response to triggers (e.g., shouting, verbal or physical aggression). Each subscale consists of five items rated from 0 to 10, with higher scores indicating greater severity of misophonia symptoms. In the present study, Cronbach’s alpha (α) for the total score was 0.91, while subscale alphas in the validation study ranged from 0.81 to 0.92 (53).
9. Treatment satisfaction was measured by asking participants in the post-treatment online assessment: “Keeping in mind that cognitive reappraisal is only one of many therapeutic methods, would you recommend this method (as one of various methods) to a friend?”. Responses were given on a 5-point scale: “I would definitely discourage,” “I would rather discourage,” “I would neither recommend nor discourage”, “I would rather recommend,” and “I would definitely recommend”.
10. Focus groups. To explore the lived experiences of the participants, two focus groups were audio-recorded. Volunteering participants (*N* = 11) shared their experiences. Participants were encouraged to speak, but could choose not to participate, and some had their cameras switched off. Throughout the study, we did not collect identifiable data and used codes to identify participants and match the data.

Before the interviews, a list of main questions was prepared: What was the most valuable part of the project? What was the most challenging part of the project? What types of CR were the most and least helpful? At what point in time did you find CR most adequate (before, during, after the trigger)? The interviewer(s) engaged in the conversations, commenting or answering questions, asking for clarifications, and posing additional questions. Because one of the most important goals of this pilot study was to verify the needs of people with misophonia, assess treatment satisfaction, and further modify the protocol, we encouraged participants to share their struggles, potential deteriorations, and other negative aspects of the therapy. Examples of the comments and questions we asked were as follows: “If we were to write a manual for us and for other therapists, what should we know about misophonia? Does anything come to mind? [silence] What should such a therapist have written in the manual, maybe even highlighted in red? [silence] What should they say, what should they not say, what should they avoid”; “Was there anything in the group that was an obstacle, or that made things harder?”. After initial reflections on difficulties emerged, the interviewer responded: “Thank you for sharing this. In a moment, if I may, I’d like to ask a few more questions., but I wonder if anyone else had similar feelings?” or “I will return to Ms. T shortly, and if I may, I would like to ask about Ms. S and Ms. B (initials changed). Do you have any insight into what made these sessions so difficult? Could they have been different?”

Although the interviewers took on clear roles as experts/researchers, the aim of this group meeting was to create an atmosphere of collaboration, genuine interest in the participants’ experiences, and the exchange of ideas, rather than a rigid interview where the researcher maintains detachment from the participants.

Being aware of the impact of our personal experiences on data interpretation and meaning-making, in accordance with Braun and Clarke (39, 40)⁠, we reveal the following factors that might be significant: MS: One of the psychotherapists (in cognitive behavioral therapy training) and researchers meeting the participants; personal experience with misophonia that the participants were aware of; AT: One of the psychotherapists (in CBT training) and researchers meeting the participants through the whole study. WA: One of the researchers, who met and assessed the participants, did not participate in the interviews; a 3rd year psychology student. MG: Scientist and psychotherapist with a background in systemic, humanistic, and mentalization-based training; did not meet the participants.

### 2.4 Intervention

The intervention consisted of four online sessions: one group session (90 minutes) and three individual sessions (30 minutes each). Full protocols will be published separately and are available from the authors upon reasonable request. Here we summarize the main structure. These materials were designed for research purposes to ensure consistency and replicability. It is not recommended to read or unreflectively follow the script in a clinical setting.

The group session (led by MS, with AT or KG as co-therapists) introduced the goals of the treatment and the principles of cognitive reappraisal (CR), motivated participants to use CR, and provided psychoeducation on misophonia and the role of psychological methods in its treatment. Misophonia-related thoughts (e.g., “He eats like a pig,” “She is rude”) were normalized, and the reciprocal relationship between thoughts and emotions was discussed. Two CR strategies were then practiced: *distancing* (e.g., focusing on facts, using humor or absurdity, or applying temporal framing) and *reframing* (e.g., generating alternative, less threatening interpretations of ambiguous or misophonic situations). Participants practiced these strategies on both every day and misophonia-specific examples and created their own reinterpretations. At the end, they received a summary “cheat sheet” for daily practice, and a more detailed guide with additional instructions was sent to them after the session.

The three individual sessions (delivered by MS, AT, KG, or AŚ) focused on tailoring CR to each participant’s experiences. Therapists worked with participants to map their individual reaction patterns using a structured table linking thoughts, emotions, physiology, attention, and behaviors before, during, and after exposure to triggers. This helped identify where reinterpretation could be introduced most effectively. Sessions included guided practice, role-plays, and “dialogues with the misophonic voice.” Realistic reinterpretations were emphasized, and motivational strategies were used to support engagement. Participants were encouraged to apply CR in varied real-life contexts (e.g., family meals, public transport, workplace settings) and to reflect on outcomes.

### 2.5 Data analysis

#### 2.5.1 Qualitative data

The data were analyzed with a reflexive thematic analysis, using a phenomenologically oriented, inductive, latent approach. In general, we aimed to flexibly follow the six-phase guide by Braun and Clarke (38–40).

The initial versions of the transcriptions were made using Transcriptor.pl. They were then reviewed and corrected. The transcriptions were translated into English using ChatGPT and further verified by two English speaking members of our team (with the aim of publishing in English). Nonetheless, the analysis was performed on the data in its original language (Polish). Firstly, individually, we familiarized ourselves with the data by reading and listening to the audio recordings. Afterward, each of us separately suggested codes and themes. All the ideas were further discussed during a group online meeting (MS, AT, WA, MG) and refined, leading to the final version. The draft of the report and discussion was made by MS and yet modified after another discussion with all the authors.

#### 2.5.2 Preliminary clinical outcomes

The study was conducted using the statistical software RStudio (Version: 2024.04.2 + 764) with the packages lme4 (54), lmerTest (55), tidyverse (56), performance (57), and ggplot2 (58) for creating graphs. Although the sample size for the upcoming fully powered RCT was already established, we aimed to recruit a minimum of 21 participants for this pilot study based on an initial power estimation calculated using the pwr package (59; k = 3; f = 0.4; sig.level = 0.05; power = 0.8). This sample size was chosen to ensure we had a sufficient number of participants to achieve qualitative data saturation during focus groups, and to reliably observe preliminary clinical trends. To evaluate the preliminary outcomes of the intervention, an analysis of score changes at three time points was conducted: twice before therapy and once after therapy. Data were collected from 23 participants who underwent the therapy. Of these, 21 participants provided complete data across all three time points, whereas 2 participants had missing data at the first measurement occasion (T1) for the S-Five scales, with complete data at T2 and T3. The analysis was conducted using a linear mixed-effects model (LMM), which accounted for random effects associated with individual differences among participants and fixed effects associated with the therapy time points. The model was fitted using the REML criterion and hypothesis testing was performed using Satterthwaite’s method using the *lme4* package for R. The effect size for the linear mixed-effects model (LMM) was calculated using the *performance* package, which provided the results of marginal R-squared and conditional R-squared. The former measures the proportion of variance explained by fixed effects in the model, specifically the effects associated with the time stages of measurement before and after therapy. The latter refers to the proportion of variance explained by both fixed and random effects in the model, capturing effects related to individual differences among study participants as well as the fixed effects related to therapy time stages. Since the effect size measured for the three time points does not reflect the effect size of the therapy itself, which was applied only between T2 and T3, Cohen’s *d* was additionally used to verify the effect size of the difference before and after the therapy. Cohen’s d was calculated based on the difference between post-treatment and pre-treatment scores (T3 – T2), such that reductions in symptom severity are reflected by negative values.

## 3 Results

### 3.1 Feasibility and acceptability-retention rate

A total of 26 participants enrolled in the study. Regarding study retention and treatment adherence, three participants dropped out: one prior to the initial on-site assessment (due to health issues), one after the assessment (no-show, reason unknown), and one after the first group session (no reason provided). This resulted in a high retention rate of 88.5% (23 out of 26 participants completing the study), which demonstrates the feasibility of the proposed recruitment, assessment, and intervention procedures for the upcoming RCT.

### 3.2 Treatment satisfaction

To evaluate treatment acceptability and overall satisfaction, a frequency analysis of the post-treatment survey was performed. The results demonstrated high acceptability, with the vast majority of participants indicating that they would recommend the intervention. Specifically, 56.5% (13) reported ‘I would definitely recommend’, and 34.8% (8) selected ‘I would rather recommend’. Only 8.7% (2) chose ‘I would neither recommend nor discourage’, and notably, none of the participants selected the discouraging options.

### 3.3 Qualitative analysis

Two themes were developed. The first theme, Shared Experience of Misunderstanding and Hope, depicts the common experience of isolation and misunderstanding while highlighting hope and the value of group and shared experiences. It comprises two codes: Misunderstanding and Hope and Group as a Source of Shared Experience. The second theme, The Need for Misophonia-Tailored but Individualized Therapy, reflects a clear call for the development of therapies that are flexible and adaptable to individual needs. It contains five codes: Group as a Source of Inspiration, Deterioration and Struggles, Lack of Universal Cognitive Reappraisal Strategies, Helplessness vs. Hope and Empowerment, and The Need for Misophonia-Informed Therapists. The themes, along with the codes and selected citations, are presented in **Table 2**. Here we include only examples of the participants’ voices.

**Table 2:** Themes, along with the codes and selected citations for the reflexive thematic analysis.

Themes	Subthemes	Examples of the stories; citations
1. Shared Experience of Misunderstanding and Hope	Misunderstanding and Hope	*...nobody would really understand. Despite sincere efforts, despite a psychological education, nobody will truly understand.* *… it’s more like if you tell someone, “I have misophonia, so please don’t make any sounds around me,” and they say, “Okay,” that’s good, but it doesn’t fully grasp what we feel when, for example, a trigger overwhelms us to the point where we’re about to explode or lose our mind. I feel like you need to have something similar to understand this, not necessarily a disorder, but if someone is fully healthy, they probably won’t get it.* *I have undergone various therapies. For the first time in my life, I encountered such a request and it was absolutely great. So, for that, I am very grateful because I don't come across such matters every day. The therapist was very empathetic, tried to understand, and when he said that if any sounds bothered me, I should inform him immediately, it was really spot on.* *For the first time, I met people who have the same issue as I do, and until now I somewhat denied* [hesitation] *denied the existence of the problem, downplayed it, and considered it to be foolish. When several people who, to a similar extent—well, not the same extent, but similar—treated it as a problem and it was, I don't know, an element that destroyed their lives to a greater or lesser degree, I felt like, wow, I can talk openly about this problem, and even more so—someone will understand me* [laughs]. *…group therapy, that group meeting, gave me hope, that okay, I’m not alone with this, that others have similar issues and we can support each other.* *Knowing that another person goes through similar experiences gives me hope that I'm not alone and we can support each other.* *I don't feel that I am left alone with this problem; I feel that someone is working on it. I used to always think that there was no help from anywhere.* *I kind of feel that we’re all working for a good cause, and honestly, I’m very happy that you’re doing this research. It’s very important to me, this whole experiment is very important, and frankly, I didn’t want to disrupt it by saying that I didn’t like something. I wanted to move forward faster and I didn’t want to mess up the meeting's agenda. In short, I didn’t feel like I had the space to say it, like it was a good time and moment to ruin such experiments. I didn’t want to do that at all.*
			Group as a Source of Shared Experience	*For the first time, I met people who have the same issue as I do, and until now I somewhat denied* [hesitation] *denied the existence of the problem, downplayed it, and considered it to be foolish. When several people who, to a similar extent—well, not the same extent, but similar—treated it as a problem and it was, I don't know, an element that destroyed their lives to a greater or lesser degree, I felt like, wow, I can talk openly about this problem, and even more so—someone will understand me* [laughs]. *…group therapy, that group meeting, gave me hope, that okay, I’m not alone with this, that others have similar issues and we can support each other.* *Knowing that another person goes through similar experiences gives me hope that I'm not alone and we can support each other. I wonder if, instead of having three individual sessions in a row each week, they could be interspersed with a group session where we refer to the individual sessions. Hearing someone say, "I have the same issue" or "I also struggle with this" might help me get through the tough moments.*
	2. The need for misophonia tailored but individualized therapy	Group as Source of Inspiration	*They started a discussion, and everyone could express their doubts about the overall effectiveness of this method, but it also helped me that there were people who approached it enthusiastically and very openly, and I did the same, looking at them. They kind of helped me open up to it. To approach it with less cynicism* [laughter]*. Or those on whom these techniques really work* [laughter]. *And like a friend here said, when someone says, "hey, this really worked for me." And then I'd think, "wow, if it worked for this person, maybe I should open up more and it will work for me too." Otherwise, I was more discouraged by my own failures and kind of closed off.* *And people also have cool ideas, for example, on reinterpretation, so you can always get inspired.* *Group therapy also allowed me to not always speak up, but to learn from other people's comments, mistakes, and interpretations.* *I struggled with reinterpretation. I also had a problem because I struggled with it. And probably, when someone else, like you ladies here, say they had similar experiences, it makes me feel a bit better.* *That could also be valuable for therapists, if, for example, halfway through, after two sessions, there was a group meeting with feedback going both ways.*
Deterioration and Struggles		*I struggled with reinterpretation. I also had a problem because I struggled with it. And probably, when someone else, like you ladies here, say they had similar experiences, it makes me feel a bit better.* *I'll be the most negative here because, for me, these techniques didn't help at all. I didn't see any of them working for me. Some of you mentioned that a certain persistence made things easier. For me, these techniques just made me analyze the situation even more and focus on it even more.* *…when discussing misophonia and triggers and having to recall the situation I was in—there was an exercise where I had to put myself in a really horrible situation—my thinking gets muddled, and the emotions take over. That’s why it can be difficult. Maybe a brief session, like 15 minutes, just talking about emotions and thoughts and what they are and why, might help.* *I felt like I was constantly talking about the same thing, always dealing with my emotions, which were not emotions but thoughts, or not thoughts but emotions, the definition of it. I was very, very tired…* *Well, the chart was difficult for me too. Especially when I had to identify my thoughts* [pause] *The thoughts I have during the sound. For me, it's more about emotions, and those thoughts are like... I was saying, "Oh my God, something." It felt a bit forced, but it's true, I do think that way, but I don't focus too much on what I'm thinking. Those thoughts aren't fully formed. It was hard for me.* [about the most challenging parts] *I think naming the emotions that were there because it was quite difficult to think "what is it" or realize what emotions were lying dormant because they were mostly unnamed. They were just there, but…* *The chart allowed each irritating sound to be broken down into its components.* *I felt like we were following the protocol too strictly and had little time for our personal reflections on the sounds.* *It was related to that table* [about the Table from Appendix 2]*. I don't remember exactly, but it was definitely about questions like: whether something happens before, whether I notice, whether I listen, whether I sense that sounds will occur when I see a plate of food or a person walking with food. Things like that. I never had that because my brain just keeps hoping until the last moment that there won't be any sounds. And when that changed, my quality of life drastically, drastically, drastically decreased.* *I also have a very analytical mind, so I think that contributed to my focus being 100% on the sounds. This caused me to break everything down into its components, as mentioned here, and it intensified negative emotions for me. Fortunately, I've managed to push that aside now. I don't go around seeking out sounds anymore.*
No Universal Type of Cognitive Reappraisal		*For example, humor helps me a lot, and it helps me with interpreting, reinterpretation of the sound source. And best of all, when I told my husband that I imagined him eating a carrot as if he were a horse, he started laughing so much that it completely changed the situation.* *I don’t approach this with humor at all. I can’t turn it off to come up with something like that. So I can’t do it. For me, it would be the hardest.* *I had it in such a way that when it came to time, it worked, but only in a very narrow range of situations where I felt that it would really end soon. Then I could tell myself that it would be over soon, and it was easier for me.* *It didn’t work for me at all that it’s a time-limited situation. Because my brain immediately suggests, okay, but this will happen many more times in your life, so don’t rejoice that it will end now.* *It doesn’t work for me in terms of time at all. To realize that it’s only for a moment, no, I’m just waiting then. And waiting, and it builds up, and I keep waiting.*
			Helplessness vs. Hope and Empowerment	*But it made me realize that I can work on myself and do something about it, or just wear myself out or shut myself at home.* *Well, for example, I realized that I can change my thinking about sounds. And yes, it requires work, but it is possible. I also manage to avoid less and less, not being afraid to face the sounds. It depends a lot on my disposition on a given day and at a given moment, but it is possible*
Need for Misophonia Informed Therapists		*Well, I once went to a psychotherapist specifically because of misophonia.* [...]*. And she just started digging into: when it started, what my family life was like back then. And I didn't like that very much, because the therapist suggested such an unfair idea, in my opinion. [pause] But I think it was simply due to a lack of knowledge on the subject.* [...] *That it had to do with my choices regarding* [removed due to data anonymization]*. It was really far-fetched* *I have undergone various therapies. For the first time in my life, I encountered such a request and it was absolutely great. So, for that, I am very grateful because I don't come across such matters every day. The therapist was very empathetic, tried to understand, and when he said that if any sounds bothered me, I should inform him immediately, it was really spot on.* *I wonder if, instead of having three individual sessions in a row each week, they could be interspersed with a group session where we refer to the individual sessions. Hearing someone say, "I have the same issue" or "I also struggle with this" might help me get through the tough moments.*

#### 3.3.1 Shared experience of misunderstanding and hope

***Misunderstanding and Hope*** Although we did not ask directly about this, a common sentiment of feeling misunderstood, helpless, or ‘different’ could be heard:

…nobody would really understand. Despite sincere efforts, despite a psychological education, nobody will truly understand.

It was also noted that the feeling of being heard and understood was a significant part of the therapeutic relationship:

pt?>I have undergone various therapies. For the first time in my life, I encountered such a request and it was absolutely great. So, for that, I am very grateful because I don’t come across such matters every day. The therapist was very empathetic, tried to understand, and when he said that if any sounds bothered me, I should inform him immediately, it was really spot on.

The importance of the group sessions was one of the most widely discussed topics during the interviews, an experience that allowed our participants to see that there are other people with similar problems, some of whom share the experience of misophonia.

Additionally, the awareness that there are researchers studying misophonia was highlighted as important for the participants:

I don’t feel that I am left alone with this problem; I feel that someone is working on it. I used to always think that there was no help from anywhere.

Nonetheless, this awareness also had a negative side. When asked about the opportunity to discuss their experienced struggles, one of the participants said:

I kind of feel that we’re all working for a good cause, and honestly, I’m very happy that you’re doing this research. It’s very important to me, this whole experiment is very important, and frankly, I didn’t want to disrupt it by saying that I didn’t like something. I wanted to move forward faster and I didn’t want to mess up the meeting’s agenda. In short, I didn’t feel like I had the space to say it, like it was a good time and moment to ruin such experiments. I didn’t want to do that at all.

***Group as a Source of Shared Experience*** For many of our participants, it was the first time they spoke openly about their misophonia or met other misophonia sufferers:

…group therapy, that group meeting, gave me hope, that okay, I’m not alone with this, that others have similar issues and we can support each other.

#### 3.3.2 The need for misophonia-tailored but individualized therapy

***Group as a Source of Inspiration*** As mentioned above, the group meeting was an important part of the project. Notably, it was not only in terms of not feeling alone but also as a source of inspiration and motivation. The attitude of some participants could also encourage those who were more skeptical:

They started a discussion, and everyone could express their doubts about the overall effectiveness of this method, but it also helped me that there were people who approached it enthusiastically and very openly, and I did the same, looking at them. They kind of helped me open up to it. To approach it with less cynicism [laughter]. Or those on whom these techniques really work [laughter].

The group discussion would also serve as a source of ideas:

And people also have cool ideas, for example, on reinterpretation, so you can always get inspired.

There was even an interesting suggestion made by one of the participants for further study: individual sessions could be interspersed with group sessions to boost motivation, normalize potential struggles, and, consequently, facilitate change:

I wonder if, instead of having three individual sessions in a row each week, they could be interspersed with a group session where we refer to the individual sessions. Hearing someone say, “I have the same issue” or “I also struggle with this” might help me get through the tough moments.

It could also help participants to openly speak about their experiences with the therapist and, as noted, it could also be beneficial for the therapist:

That could also be valuable for therapists, if, for example, halfway through, after two sessions, there was a group meeting with feedback going both ways.

The topic of the therapist’s knowledge about misophonia was also raised, highlighting the importance of receiving suitable treatment and the need for therapists to have proper, updated, knowledge of misophonia:

Well, I once went to a psychotherapist specifically because of misophonia. [ … ]. And she just started digging into: when it started, what my family life was like back then. And I didn’t like that very much, because the therapist suggested such an unfair idea, in my opinion. [pause] But I think it was simply due to a lack of knowledge on the subject. [ … ] That it had to do with my choices regarding [removed due to data anonymization]. It was really far-fetched.

***Helplessness vs. Hope and Empowerment*** Another aspect of the therapy that was mentioned by the participants is the sense of empowerment and the feeling that the person is not helpless. The feeling that there are things they can do, even though there is no cure for misophonia yet.

Well, for example, I realized that I can change my thinking about sounds. And yes, it requires work, but it is possible. I also manage to avoid less and less, not being afraid to face the sounds. It depends a lot on my disposition on a given day and at a given moment, but it is possible.

But it made me realize that I can work on myself and do something about it, or just wear myself out or shut myself at home.

***Deterioration and Struggles*** On the other hand, there were also participants who found the treatment challenging or who did not find the proposed techniques useful:

I’ll be the most negative here because, for me, these techniques didn’t help at all. I didn’t see any of them working for me. Some of you mentioned that a certain persistence made things easier. For me, these techniques just made me analyze the situation even more and focus on it even more.

For some people, even speaking about their misophonic experiences during the session could be so overwhelming that they are not able to fully engage in practicing reappraisals:

…when discussing misophonia and triggers and having to recall the situation I was in—there was an exercise where I had to put myself in a really horrible situation—my thinking gets muddled, and the emotions take over. That’s why it can be difficult. Maybe a brief session, like 15 minutes, just talking about emotions and thoughts and what they are and why, might help.

Many participants pointed out the difficulties in disentangling thoughts and emotions, stressing the need for a very individualized approach and therapy pace:

Well, the chart was difficult for me too. Especially when I had to identify my thoughts … [pause] The thoughts I have during the sound. For me, it’s more about emotions, and those thoughts are like … I was saying, “Oh my God, something.” It felt a bit forced, but it’s true, I do think that way, but I don’t focus too much on what I’m thinking. Those thoughts aren’t fully formed. It was hard for me.

It was noted that a group could also help to normalize the fact that the proposed techniques may not be easy immediately and may be frustrating:

I struggled with reinterpretation. I also had a problem because I struggled with it. And probably, when someone else, like you ladies here, say they had similar experiences, it makes me feel a bit better.

Nonetheless, despite the struggles, the importance and value of breaking the misophonia experience into pieces was noted:

[about the most challenging parts] I think naming the emotions that were there because it was quite difficult to think “what is it” or realize what emotions were lying dormant because they were mostly unnamed. They were just there, but…

The specificity of the research project did not allow for full adjustment to individual needs, which was also evident in participants’ accounts:

I felt like we were following the protocol too strictly and had little time for our personal reflections on the sounds.

Discussing the interplay of thoughts, emotions, physiology, attention, and behavior before, during, and after the trigger was challenging for some participants:

It was related to that table…. I don’t remember exactly, but it was definitely about questions like: whether something happens before, whether I notice, whether I listen, whether I sense that sounds will occur when I see a plate of food or a person walking with food. Things like that. I never had that because my brain just keeps hoping until the last moment that there won’t be any sounds. And when that changed, my quality of life drastically, drastically, drastically decreased.

***No Universal Type of Cognitive Reappraisal*** Regarding the main topic of this project - it seems there is no common ideal type of CR nor an optimal time to use it. It may all depend on the individual needs and experience of the person, as well as the type of trigger and situation. For example, some of the participants shared a very positive experience with using humor as a method for reinterpretation:

For example, humor helps me a lot, and it helps me with interpreting, reinterpretation of the sound source. And best of all, when I told my husband that I imagined him eating a carrot as if he were a horse, he started laughing so much that it completely changed the situation.

While others found it the most difficult or inappropriate:

I don’t approach this with humor at all. I can’t turn it off to come up with something like that. So I can’t do it. For me, it would be the hardest.

Regarding focusing on time as a form of distancing, it tended to be helpful in some types of misophonic situations for some participants:

I had it in such a way that when it came to time, it worked, but only in a very narrow range of situations where I felt that it would really end soon. Then I could tell myself that it would be over soon, and it was easier for me.

While others struggled with using it:

It didn’t work for me at all that it’s a time-limited situation. Because my brain immediately suggests, okay, but this will happen many more times in your life, so don’t rejoice that it will end now.

It doesn’t work for me in terms of time at all. To realize that it’s only for a moment, no, I’m just waiting then. And waiting, and it builds up, and I keep waiting.

Although misophonia sufferers share common experiences, their responses to treatment varied considerably.

### 3.4 Protocol refinements based on feasibility findings

In line with methodological frameworks for pilot studies, which emphasize identifying necessary protocol modifications before a definitive trial (44, 45), qualitative feedback from participants provided crucial insights for refining the intervention protocol prior to the main RCT. Based on the reported difficulties and feelings of exhaustion (e.g., struggles with distinguishing thoughts from emotions and analyzing the reaction patterns table), the protocol for the main trial was modified to allow for greater flexibility and individualized pacing. Specifically, the emphasis on rigorously completing all sections of the reaction patterns table during the session was reduced; therapists now encourage finishing it at home if needed. Furthermore, therapists were instructed to emphasize respecting participants’ boundaries by ensuring they are ready to discuss their misophonic experiences, and checking whether such discussions might evoke overly intense emotions at that time. Additionally, participants are explicitly guided to express when they consider reinterpretation, or any other part of the protocol, to be inappropriate for them.

To address feelings of frustration when reappraisal did not yield immediate relief, we added specific discussions regarding expectation management. Therapists now explicitly clarify the differences between avoidance strategies (which are widely known to misophonia sufferers and may produce immediate symptom disappearance) and reinterpretation (which does not usually produce such spectacular, immediate results, but prevents long-term losses). A reality check was integrated into the protocol to ensure participants do not expect their symptoms to disappear entirely. This is reinforced in the third session using Socratic dialogue to remind participants of their therapeutic goals and to address potential thoughts of “this isn’t working” and their subsequent impact on emotions. It is also emphasized that reinterpretations cannot be too far from what the individual genuinely believes, as they may otherwise lose their effectiveness.

Additionally, recognizing that deeply ingrained convictions related to misophonia (e.g., “people shouldn’t eat like that” or “this IS disgusting”) are often extremely difficult to change, we refined the instructions for “dialogues with the misophonic voice.” The revised strategy clarifies that CR is not always about questioning the reality of thoughts or “facts” (e.g., discussing whether someone will actually laugh at them for asking to stop slurping, or whether a behavior is objectively polite or not). Instead, it focuses on how fixating on these statements negatively affects emotions, physiology, and attention. To illustrate this, we introduced the “wheelchair metaphor” during individual sessions. This metaphor demonstrates that just as telling a person who recently started using a wheelchair “you can’t even make tea” is a hurtful statement of fact, labeling a sound as “disgusting” or “impolite” might be perceived as a fact, but only serves to elicit strong negative feelings. Therefore, rather than arguing with the reality of the thought, participants are encouraged to use cognitive distancing and reinterpretation (e.g., “it will soon pass” or “it sounds like a boot splashing in a puddle”) or acceptance (e.g., “ok, it is disgusting, but it doesn’t make sense to ruminate on it” or “my thinking about it will not change their behavior”) to reduce negative feelings and improve their current situation.

Finally, to address concerns raised by some individuals about “ruining the experiment”, a specific instruction was added to the group session explicitly encouraging participants to openly share their struggles and negative experiences. It was emphasized that they do not need to please the researchers or force positive responses for the study to “succeed,” which aims to reduce social desirability bias in the upcoming trial.

### 3.5 Preliminary clinical outcomes

**Figure 2:**
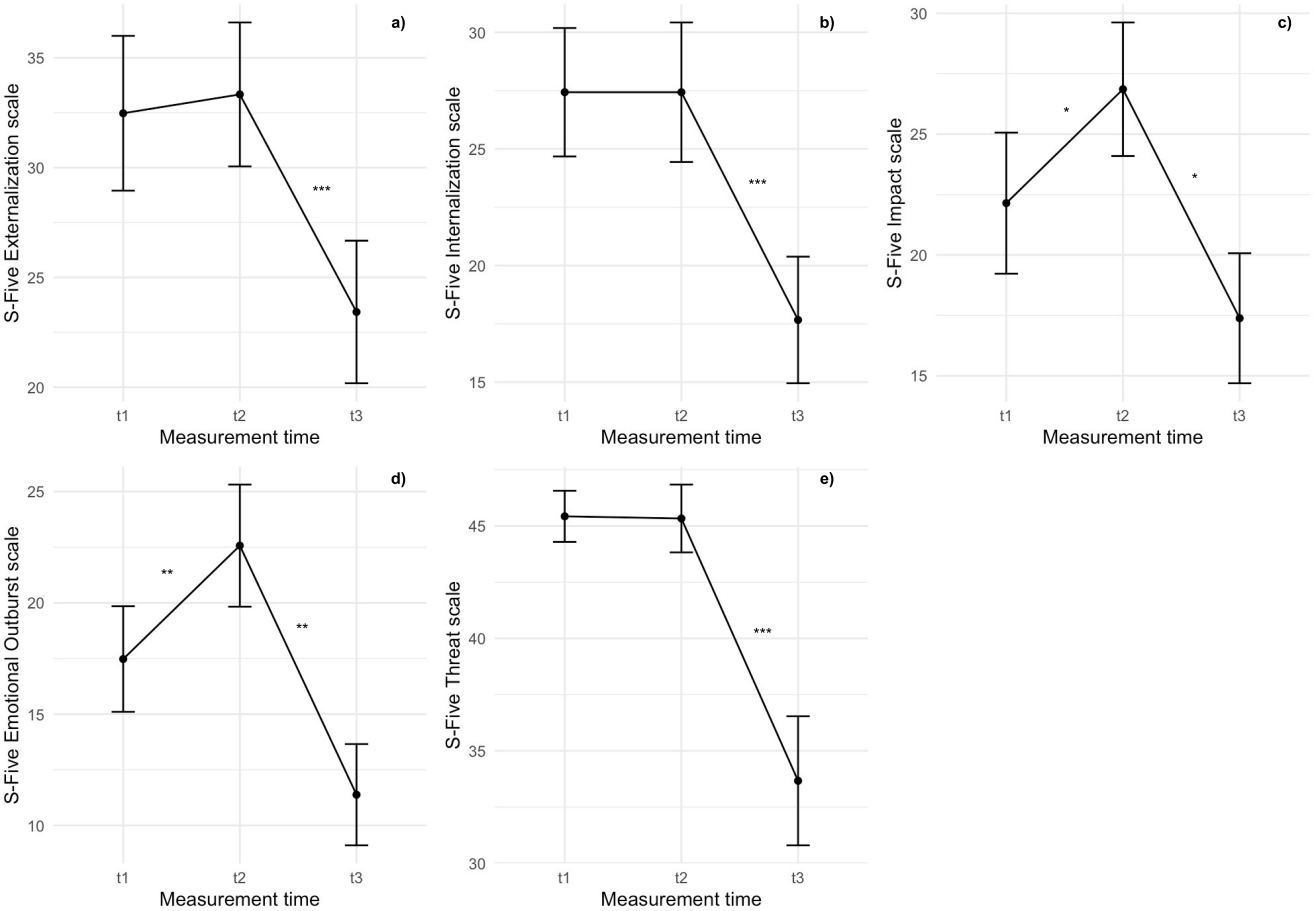
Changes across measurement time points (t1–t3) for the S-Five subscales: **(a)** Externalization, **(b)** Internalization, **(c)** Impact, **(d)** Emotional Outburst, and **(e)** Threat. Error bars represent 95% confidence intervals. Statistical significance is indicated as follows: * p < .05; ** p < .01; *** p < .001.

The first step in verifying the initial outcomes of CR was to analyze changes on the S-Five scale using linear mixed-effects models (LMM). Outcomes were assessed at three time points: T1 (initial screening), T2 (pre-treatment), and T3 (post-treatment). The inclusion of three measurements allowed us to control for potential confounding factors: stability was expected between T1 and T2, whereas significant change was hypothesized between T2 and T3 as an effect of the intervention. The fixed effect estimates, along with standardized effect sizes (Cohen’s d) and their 95% confidence intervals for all S-Five subscales (negative values indicate symptom reduction), are systematically summarized in **Table 3**. The results (see **Figures 2A–E**) were analyzed separately for each S-Five subscale.

**Table 3:** Changes in S-Five subscales across waiting and treatment phases.

S-Five subscale	Waiting period (T1–T2) estimate (*SE*)	*p*	Treatment phase (T2–T3) estimate (*SE*)	*p*	Cohen’s *d* [95% CI]
Externalizing appraisals	0.23 (2.32)	.920	-8.47 (2.32)	< .001	-0.76 [-1.22, -0.29]
Internalizing appraisals	0.02 (2.15)	.990	-9.50 (2.15)	< .001	-0.91 [-1.39, -0.41]
Emotional outburst	5.47 (2.33)	.024	-5.96 (2.33)	.014	-0.89 [-1.36, -0.39]
Impact	4.95 (1.76)	.007	-5.01 (1.76)	.007	-1.03 [-1.53, -0.52]
Threat	0.16 (2.39)	.950	-11.58 (2.39)	< .001	-0.86 [-1.34, -0.37]

#### 3.5.1 Externalizing appraisals

For the Externalizing appraisals subscale, the mean score at T1 was 32.34 (*SE* = 3.16), and at T2 it was 32.56, showing no significant difference between these two time points. In contrast, the mean score at T3 decreased to 23.87, representing a significant reduction compared to both T1 and T2. The effect size for this change between T2 and T3, calculated with Cohen’s *d* for paired samples, was -0.76 (95% CI [-1.22, -0.29]), indicating a medium to large effect size for the change over time. Variability among participants was substantial (*variance* = 164.12), suggesting that the CR intervention did not benefit all individuals equally.

#### 3.5.2 Internalizing appraisals

The results for the Internalizing appraisals subscale followed a similar pattern. The mean score at T1 was 28.07 (*SE* = 2.72), and there was no significant difference between T1 and T2, indicating stability during the waiting period. In contrast, a significant reduction was observed at T3 compared to both T1 and T2. Because T1 and T2 did not differ, the contrasts T1–T3 and T2–T3 produced identical results. The effect size for the change between T2 and T3, calculated with Cohen’s *d* for paired samples, was -0.91 [95% CI (-1.39, -0.41)], indicating a large effect size for the change over time. Variability among participants was again substantial (*variance* = 113.11). These results suggest that CR substantially reduced unhelpful internalizing appraisals, although the magnitude of improvement varied across individuals. Results for both the externalizing and internalizing subscales are presented in **Figures 2A, B**.

#### 3.5.3 Emotional outburst

For the Emotional outburst subscale, the mean score at T1 was 18.05 (*SE* = 2.41). Scores worsened significantly between T1 and T2, which indicated deterioration during the waiting period. In contrast, at T3 there was a significant reduction relative to T1. Consistent with this pattern, the pre- to post-treatment change (T2–T3) showed a large effect (Cohen’s *d* = -0.89, 95% CI [-1.36, -0.39]). Variability between participants remained substantial (random-intercept variance = 66.69), though it was smaller than for the Externalizing and Internalizing subscales.

#### 3.5.4 Impact

For the Impact on daily functioning subscale, the mean score at T1 was 22.10 (*SE* = 2.63). Scores worsened significantly between T1 and T2, indicating increased functional impairment during the waiting period. In contrast, at T3 there was a significant reduction relative to T1, with participants’ scores after therapy being significantly lower than at both T1 and T2. The effect size for the T2–T3 change, calculated with Cohen’s d for paired samples, was -1.03 (95% CI [-1.53, -0.52]), indicating a very large effect size for the change over time. Variability between participants was high (*variance* = 121.15), comparable to the Externalizing and Internalizing subscales. Results for both the Emotional outbursts and Impact subscales are presented in **Figures 2C, D**.

#### 3.5.5 Threat

For the Threat subscale, which measures distress about emotions escalating around triggering sounds, the mean score at T1 was 45.58 (*SE* = 1.98). There was no significant difference between T1 and T2, indicating stability during the waiting period. In contrast, a significant reduction was observed at T3 compared to both T1 and T2. The effect size for the change between T2 and T3, calculated with Cohen’s *d* for paired samples, was -0.86 (95% CI [-1.34, -0.37]), indicating a large effect size for the change over time. Variability between participants was the lowest among all subscales (*variance* = 21.07), suggesting that the majority of individuals showed a similar pattern of improvement. Results for this subscale are presented in **Figure 2E**.

**Figure 3:**
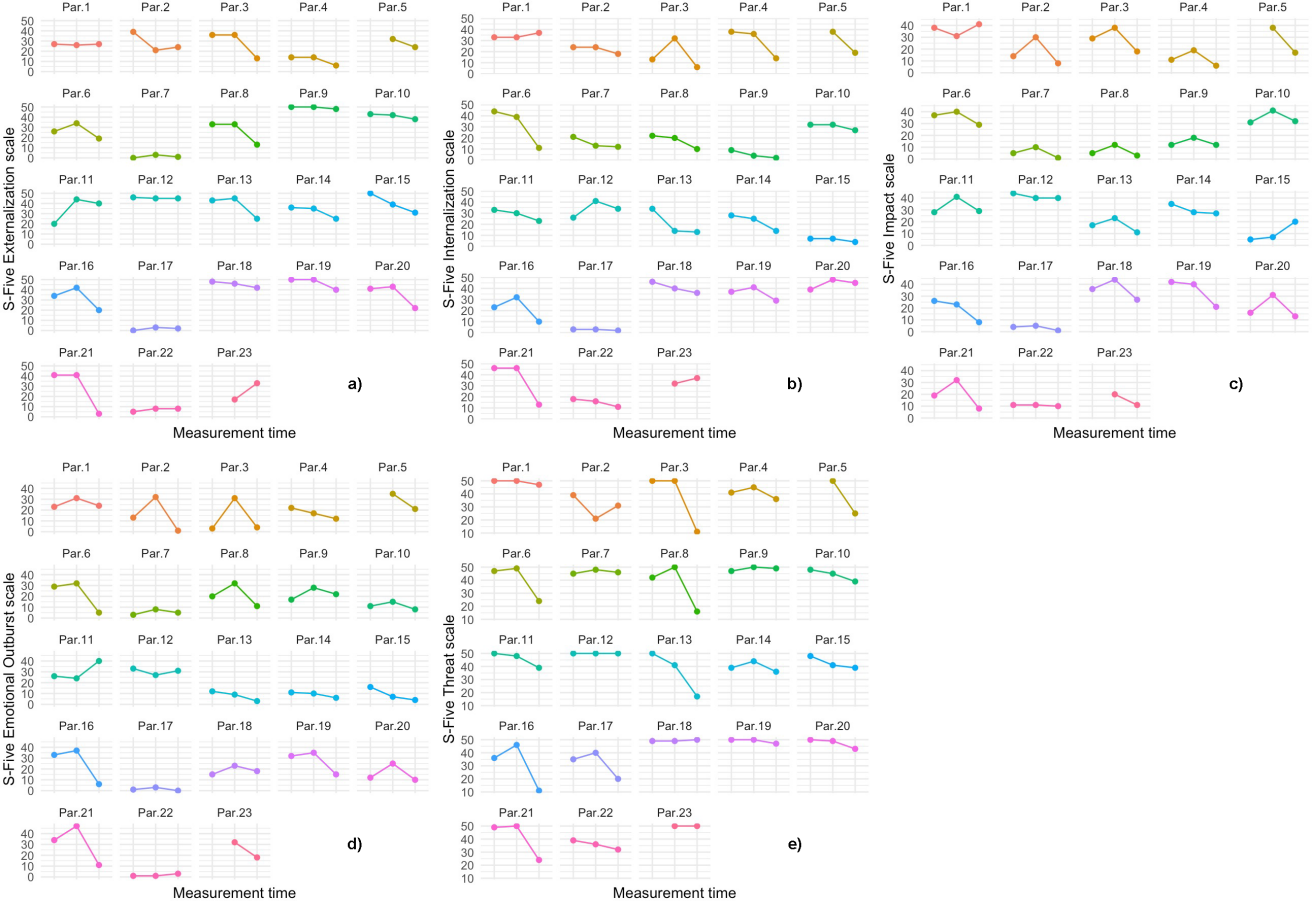
Average scores for each S-Five scale across three time intervals for each individual. Individual trajectories of participants across measurement time points (t1–t3) for the S-Five subscales: **(a)** Externalization, **(b)** Internalization, **(c)** Impact, **(d)** Emotional Outburst, and **(e)** Threat. Each small panel represents one participant and illustrates changes in scores over the course of the intervention.

Given the considerable variability among study participants and the small sample size, we complemented the group-level analyses with plots showing the trajectory of each individual separately. These visualizations make it possible to identify participants who improved, those who remained stable, and those whose scores worsened despite the overall positive group effect. Presenting these individual patterns provides a bridge between the quantitative and qualitative parts of the study, as it highlights the need to explore the subjective experiences that may explain such heterogeneous outcomes. The plots for all subscales are presented in **Figure 3**.

#### 3.5.6 Number of responders

Lastly, we calculated the number of responders using the minimal clinically important difference (MCID; 60). Due to missing data for two participants we only calculated the number of responders for 21 participants. The half-standard deviation (0.5 SD) thresholds for the subscales were as follows: Externalizing Appraisals: 8, Internalizing Appraisals: 6, Outburst: 5, Impact: 7, and Threat: 3.

Improvement rates were as follows: Threat: 17 (81%), Internalizing: 15 (71%), Externalizing: 11 (52%), Outburst: 10 (48%), and Impact: 7 (33%).

It is also worth noting the number of participants who deteriorated, using the same 0.5 SD measure: Internalizing: 2 participants, Externalizing: 1 participant, Outburst: 2 participants, Threat: none, and Impact: 1 participant.

## 4 Discussion

This pilot study aimed to evaluate, consistent with current conceptual frameworks for pilot studies (44, 45), the feasibility, acceptability, and preliminary outcomes of a 4-week cognitive reappraisal (CR; 22, 24) intervention for misophonia, providing early, practice-based data from a psychotherapy-like, non-laboratory setting. Our pilot is best positioned not as an evaluation of a candidate stand-alone treatment, but rather as an investigation of CR as one specific, active component that can be integrated within the broader family of process-based cognitive behavioral therapies.We also explored participants’ lived experiences, with Reflexive Thematic Analysis, including the challenges they encountered during the treatment, in order to modify the protocols for the RTC, as well as the satisfaction from the treatment. As a result, we revised the study protocol in order to make the study procedures and CR intervention more accessible and better tailored to the needs of individuals with misophonia (the protocols is available upon reasonable request). We also assessed the primary outcomes of the intervention. Regarding our primary pilot objectives, treatment satisfaction was remarkably high, and the dropout rate was very low, with 88.5% (23 out of 26) of enrolled participants completing the study. These findings strongly indicate the feasibility and overall acceptability of the proposed intervention procedures. Furthermore, while the quantitative data should be interpreted cautiously as preliminary trends, the initial clinical signals are highly encouraging. Notably, the majority of participants achieved a clinically meaningful improvement (based on MCID) in three out of the five misophonia domains measured by the S-Five scale. At the same time, the percentage of individuals reporting any symptom worsening was very small, and crucially, no single participant deteriorated across all aspects of misophonia.

Both themes developed through Reflexive Thematic Analysis highlighted the significance of the group sessions, which were initially designed as a practical and efficient method for delivering CR, rather than as a direct source of change, through mechanisms related to social support, shared experience or feelings’ validation. However, participants shared that the group setting not only provided a platform to exchange experiences and connect with others facing similar challenges but was also seen as a source of inspiration and motivation. Given the common feelings of isolation, difference, and misunderstanding experienced by individuals with misophonia.

Challenges during individual therapy sessions emerged as a significant focus in the interviews as well. Some participants reported feelings of exhaustion and frustration due to difficulties in differentiating between thoughts and emotions. They expressed a need for more time not only to discuss reappraisals but also to address their uncertainties. It is noteworthy that the individual sessions (30 minutes) and overall treatment duration (four sessions) were shorter than typical psychotherapy, which may have limited participants’ opportunities to explore thoughts and emotions in depth. With more time, it is likely that patients and therapists would be able to talk much more about differences between thoughts and feelings, patterns of thoughts, and other similar topics. It was also noted that discussing misophonic experiences could be too stressful for some individuals, which might hinder their ability to distinguish between thoughts and emotions during the sessions, making it less beneficial. These could be potential factors explaining the worsening of misophonia symptoms that should be explored in future studies. However, a recent study showed the opposite effects, indicating that self-efficacy, neuroticism, emotional identification and stress were moderators of outcomes, for example with higher level of neuroticism or lower level of self-efficacy were associated with better outcomes (61)⁠. Thus, the interplay of these factors may be more complex.

In our study, we introduced participants to selected types of CR, including reframing (finding another perspective or broadening the context) and distancing (observing like a reporter, focusing on time, or using humor or absurdity). We observed that there is no one-size-fits-all approach to CR. Its effectiveness may depend not only on individual preferences and abilities but also on the person’s current state and the types of triggers involved. Future studies should not only explore the most effective forms of CR but also assess how the acceptance of specific CR techniques contributes to improvement. Previous research has shown that CR can be maladaptive in controllable situations (62)⁠. In the context of misophonia, this suggests that individuals might benefit from not overly engaging in CR when the trigger can be avoided without significant consequences, such as social withdrawal.

Here, it is also worth noting that in clinical practice, it is not always possible to draw a strict line between cognitive reappraisal as a ‘classical’ CBT technique and interventions rooted in Acceptance and Commitment Therapy (ACT). For example, after the pilot study, in the second individual session, we introduced the metaphor of a person in a wheelchair (the protocol with changes after the pilot study will be published in a separate publication from the ongoing RCT). This metaphor aimed to illustrate that even if a thought is objectively true—or if we strongly believe it to be true—repeatedly focusing on it may not be beneficial. As such, rather than relying solely on thought modification, we utilized cognitive distancing through acceptance and directed participants’ attention toward their personal goals and values.

An important message for therapists working with individuals with misophonia, particularly in light of the limited knowledge regarding the mechanisms of misophonia and the findings from previously mentioned studies (61–63), is that, in certain situations, CR is not always be the best or first option for addressing misophonic symptoms. Instead, emphasis may be placed initially on creating a safe environment for participants to practice CR in less distressing, non-misophonic contexts. Other established CBT processes, such as attention-shifting. mimicking—an method intuitively practiced by many of our participants, psychoeducation, inhibitory learning, or even avoidance strategies like using headphones, may also be worth incorporating. All these techniques, including CR, however, should be explored more thoroughly in future studies and adjusted to individuals needs and specificity of misophonia.

Additionally, some participants noted that too much emphasis was placed on completing the table identifying patterns of thoughts, emotions, physiology, behavior, and attention before, during, and after triggers. This process temporarily increased frustration and distress in several individuals. It may be helpful for therapists to remember that the goal of filling out the table is not to complete every ‘cell,’ but rather to help participants identify their individual patterns of reaction and better understand their responses. For example, in some cases, individuals may not focus excessively before the trigger occurs or may not experience ‘angry’ thoughts.

While psychoeducation during group meetings on the potential mechanisms of misophonia was provided, the extensive focus on thoughts might have led some participants not only to overfocus, but also to believe that misophonia solely arises from their thoughts. Although we developed the study from the broader framework of a process-based therapy approach (23), the specificity of the procedures of the research project might have impacted the experience.

Therapists working with misophonia sufferers are, however, not encouraged to strictly follow the proposed procedures or attached protocol. In this project, we focused solely on one of many techniques that could or should be used in misophonia treatment, and these methods should be flexibly adapted to each client’s needs in a clinical setting—something that, despite our best efforts, is not entirely achievable in a research study. It is important to remember that while the context and cognitive aspects may play a significant role in misophonia (64)⁠, misophonic reactions are likely not driven solely by cognitive factors. For therapists trained in CBTs, it may be instinctive to assume otherwise (65)⁠. However, throughout the therapeutic process, it is crucial to recognize that modifying thoughts alone will not eliminate the misophonic reaction. Moreover, when addressing thoughts and beliefs, therapists should adhere to the latest research on misophonia and avoid interpretations suggesting that misophonia stems from trauma or family dynamics for all people. This assumption is not supported by current preliminary evidence (66) and, as noted by participants (“…I didn’t like that very much, because the therapist suggested such an unfair idea, in my opinion…”), such interpretations can be very discouraging.

Regarding preliminary clinical outcomes, as expected, the majority of participants showed significant improvement across most of misophonia domains, as measured by the S-Five (52), between pre-treatment (T1) and post-treatment (T3). While we cannot completely rule out the effects of time or regression to the mean, the absence of symptom reduction between screening (T1) and pre-treatment (T2) renders these explanations far less plausible. Nonetheless, as discussed below, we cannot rule out non-specific factors such as expectancy, therapist attention, or group-based validation. An unexpected worsening was observed in the S-Five Impact Scale and Emotional Outburst Scale between the initial measurements (T1) and pre-treatment (T2). This could be attributed to participants’ anticipatory concerns about facing their misophonic triggers during the on-site assessment and therapy, which may have heightened their symptom awareness; however, we acknowledge that this explanation remains speculative. At the same time, evidence on waiting periods suggests that symptom change on waitlists is typically small and often in the direction of slight improvement (67, 68), whereas deterioration tends to be associated with longer waits in routine services (69). In our study, the 4–6 week interval between T1 and T2 reflected scheduling rather than a formal waitlist condition; nevertheless, the worsening observed on two subscales may reflect disorder-specific anticipatory processes rather than a general waitlist effect.

As shown in **Figure 3**, while CR produced large overall improvements, individual responses varied, with some participants showing minimal change or worsening. Although CR has been shown in multiple studies to effectively reduce negative emotional arousal, recent research has highlighted its potential limitations (63)⁠. We interpret our pilot findings conservatively as preliminary evidence that CR may work for some (but not all) people with misophonia. Crucially, we do not position CR as a stand-alone intervention, but rather as one active component within broader, formulation-driven CBT therapies (e.g., 18, 35). Furthermore, to contextualize the strong, uncontrolled effect sizes observed in our study, they should be explicitly benchmarked against the rigorous RCT by Jager et al. (19). Using a waitlist control and blinded assessments, Jager et al. reported large effects (d = 1.97). It is important to emphasize that this pilot study did not include a control group or blinded assessment, making it essential to consider that our results were likely inflated by potential confounding factors, such as the positive effect of being recognized as a misophonia sufferer simply by participating in the study, or the therapeutic impact of sharing experiences in a group session.

To summarize, we examined one of the many techniques available for treating misophonia. While our findings suggest that CR is a feasible and potentially helpful technique, it should not be concluded that CR is the most important technique, nor should it be prioritized over other CBT processes based on current evidence. Instead, our study adds to the foundation laid by multi-component therapies, such as the Unified Protocol and PBT, by illuminating how one specific mechanism (CR) operates and is experienced by patients. Notably, our data, with no control group, do not allow us to determine what contributed to the decrease in misophonia symptoms, but rather let us adjust the protocol to misophonia sufferers needs and gave a rational to perform larger, randomized control trial.

It should also be stressed that our study protocol does not imply that CR is only applicable to individuals triggered by eating sounds. Indeed, during the individual sessions, whenever preferred by the participants, we also practiced applying CR to other, non-oral triggers, such as noises made by neighbors or pen clicking. As previously mentioned, the inclusion criterion requiring eating sounds was not related to the expected efficacy of CR for specific triggers, but rather to the need for standardized stimuli in the experimental component of the study (which is beyond the scope of this paper). Therefore, there is no rationale to claim that CR can only be used for eating sounds.

The therapist’s role should be to recognize individual needs and assist in selecting the most suitable and accessible types of CR or other methods. Since misophonia is a relatively new and not widely understood disorder, sufferers often feel misunderstood and may have encountered unsuccessful treatment experiences. Therefore, it is essential for therapists to be familiar with the specific nature of misophonia and to adapt commonly used techniques, such as CR, to better align with the unique characteristics of the disorder.

### 4.1 Limitations

First, we acknowledge that we did not define *a priori* progression criteria (e.g., pre-established thresholds for acceptable dropout or treatment satisfaction rates). Furthermore, because this pilot study was conducted immediately prior to the main randomized controlled trial (RCT)—which was already secured by a grant and pre-registered—it was not designed to estimate parameters for a formal sample size calculation. Instead, our primary aim was a formative evaluation to learn how to refine the existing protocol and make it more tailored to the specific needs of participants with misophonia. It should be noted that all participants were White (as non-White individuals constitute a very small proportion of the population in Poland). While this sample contributes to the heterogeneity of studies conducted primarily in the United States, it consisted predominantly of educated women, which may limit the generalizability of the findings. As this was a pilot feasibility and acceptability study, the small sample size and the lack of control group did not allow us to explore additional factors that may impact the magnitude of pre-to-post change, such as comorbid disorders, the therapeutic relationship, the effect of validation or perceived social support. Moreover, we did not control for neurodevelopmental disorders or psychotropic medication. Additionally, the short duration of the treatment may have limited the results and required an immediate focus on CR, whereas it might sometimes be more appropriate to begin with more basic or alternative interventions. This also restricted the time available for the development of the therapeutic relationship. In future studies, a follow-up assessment should also be implemented to evaluate the maintenance of treatment effects. Furthermore, excluding individuals who recently participated in psychotherapy may have biased our sample toward more treatment-naïve patients, potentially limiting the generalizability of our findings.

Another issue concerns the multiple roles of the researchers-two authors of the study (MS and AT) served in multiple roles, acting as diagnosticians, therapists, and later as interviewers in the focus groups. The close relationship with participants may have facilitated recognizing and discussing their perspectives. However, this overlap could also have prevented participants from fully sharing their experiences. Participants may have felt inhibited in voicing criticisms of the intervention during focus groups to the exact same individuals who provided their therapy. This social desirability effect could bias the qualitative data toward more positive accounts of the treatment. Nonetheless, the treatment satisfaction assessment, which was high in this sample, was completed without the presence of the researchers and reflected what we heard in the qualitative data. Future studies could mitigate this issue by involving independent researchers to conduct interviews. Finally, the effect sizes observed in this study were exceptionally large. Although T1–T2 deterioration occurred only on two of the five S-Five subscales, the T2–T3 effects were uniformly very strong across all subscales. Replication in larger, using more conservative analytic strategies, will be essential to establish the robustness of these findings.

## 5 Conclusions

In conclusion, this mixed-methods study confirmed the feasibility and high acceptability of the proposed CR procedures and helped to improve the protocol for the RCT. While preliminary quantitative trends suggest that CR holds potential as an active treatment component for misophonia, the qualitative feedback included criticism regarding some aspects of CR and its introduction. It underscored that therapists should use a highly individualized approach that balances cognitive restructuring with boundary-setting, validation, and acceptance-based strategies.

## Data Availability

The raw data supporting the conclusions of this article will be made available by the authors, without undue reservation.
